# Two-step derivatization for determination of sugar phosphates in plants by combined reversed phase chromatography/tandem mass spectrometry

**DOI:** 10.1186/s13007-019-0514-9

**Published:** 2019-11-07

**Authors:** Umut Rende, Totte Niittylä, Thomas Moritz

**Affiliations:** 1 Department of Forest Genetics and Plant Physiology, Umeå Plant Science Centre, Swedish University of Agricultural Sciences, 901 83 Umeå, Sweden; 2 The NovoNordisk Foundation Centre for Basic Metabolic Research, Faculty of Health and Medical Sciences, University of Copenhagen, 2200 Copenhagen, Denmark

**Keywords:** Sugar phosphates, UHPLC–ESI–MS, Q-TOF, QqQ-MS, Extraction, Two-step derivatization

## Abstract

**Background:**

Sugar phosphates are important intermediates of central carbon metabolism in biological systems, with roles in glycolysis, the pentose–phosphate pathway, tricarboxylic acid (TCA) cycle, and many other biosynthesis pathways. Understanding central carbon metabolism requires a simple, robust and comprehensive analytical method. However, sugar phosphates are notoriously difficult to analyze by traditional reversed phase liquid chromatography.

**Results:**

Here, we show a two-step derivatization of sugar phosphates by methoxylamine and propionic acid anhydride after chloroform/methanol (3:7) extraction from *Populus* leaf and developing wood that improves separation, identification and quantification of sugar phosphates by ultra high performance liquid chromatography–electrospray ionization–mass spectrometry (UHPLC–ESI–MS). Standard curves of authentic sugar phosphates were generated for concentrations from pg to ng/μl with a correlation coefficient *R*^2^ > 0.99. The method showed high sensitivity and repeatability with relative standard deviation (RSD) < 20% based on repeated extraction, derivatization and detection. The analytical accuracy for *Populus* leaf extracts, determined by a two-level spiking approach of selected metabolites, was 79–107%.

**Conclusion:**

The results show the reliability of combined reversed phase liquid chromatography–tandem mass spectrometry for sugar phosphate analysis and demonstrate the presence of two unknown sugar phosphates in *Populus* extracts.

## Background

Metabolomics enables comprehensive identification and quantification of small molecule metabolites that are intermediates of primary metabolic pathways, hormones and secondary metabolites in biological systems [1]. Primary metabolites, such as the intermediate metabolites of central carbon metabolism, have key functions in glycolysis, the pentose–phosphate pathway and tricarboxylic acid (TCA) cycle. These metabolites are classified as sugar phosphates, nucleotides, nucleotide sugars, carboxylic acids and phospho-carboxylic acids. Sugar phosphates act as important intermediates in energy metabolism and provide the starting point for most biosynthetic processes in prokaryotic and eukaryotic cells. In plants, the hexose-phosphate pool, consisting of interconvertible six carbon sugars glucose-6-P, glucose-1-P and fructose-6-P [2], provides carbon for cell wall and starch biosynthesis in addition to glycolysis and the pentose phosphate pathway. Hence, comprehensive analysis of plant primary metabolism requires a robust method for quantifying sugar phosphates.

Sugar phosphates are chemically unstable and sensitive to enzymatic degradation during extraction. Therefore, to obtain accurate and precise measurements of sugar phosphates in plant tissues, a rapid, simple and robust extraction method is required to avoid chemical degradation and quench enzymatic activities during sample preparation. Depending on the metabolite of interest, the most common way of avoiding chemical and enzymatic metabolite degradation during extraction is to apply low or high temperatures to homogenized plant tissues in organic solvents or a mixture of solvents [3–6]. For example, chloroform–methanol–water extraction at low temperature is suitable for extracting water-soluble and organic-soluble metabolites [6, 7]. In contrast, hot ethanol extraction is suitable for extracting polar and mildly non-polar metabolites [3, 5]. However, although this method is relatively simple, it requires consequtive ethanol extraction steps, during which some enzymes may remain active and cause changes in metabolite levels. In addition to these methods, trichloroacetic acid-ether extraction can be used to extract metabolites, but this method is only suitable for acid-stable and water-soluble metabolites [4].

Analysis of sugar phosphates is usually performed by mass spectrometry (MS) connected to capillary electrophoresis (CE), gas chromatography (GC) or liquid chromatography (LC). CE–MS has high separation efficiency and sensitivity for measuring sugar phosphates, but it requires very low injection volumes (nL) and thereby fails to detect sugar phosphates at low concentrations [8]. GC–MS analysis is suitable for identifying and quantifying volatile compounds. Hence, nonvolatile compounds, such as neutral sugars and sugar phosphates, must be derivatized prior to GC–MS analysis, e.g. by using a combination of oximation and trimethylsilylation derivatization [7]. Recently, a pentafluorobenzyl oxime and trimethylsilyl derivatization strategy combined with GC-negative chemical ionization MS showed promising results for detecting sugar phosphates in cell cultures [9]. Among combined chromatography–mass spectrometry techniques, LC–MS is commonly used for polar compounds and is compatible with aqueous phases obtained during the extraction.

Different LC–MS techniques have been developed to analyze a variety of metabolites, including sugar phosphates. For instance, ion pair chromatography (IPC), which is a type of reversed phase LC with a hydrophobic stationary phase, can be used to separate ionic compounds, such as nucleotides, sugar nucleotides and sugar phosphates. In IPC, ion-pairing reagents are added to the mobile phase to form ion pairs with ionic compounds and improve the retention and separation in the column [10–13]. However, it has been reported that IPC is not suitable for separation of isomer sugar phosphates, and removal of ion-pairing reagents from the chromatographic system is often problematic as their residues may affect MS analysis. Another approach is to combine anion chromatography (AIC) with mass spectrometry. With this approach, it is possible to separate most of the metabolites involved in glycolysis and the TCA cycle with good reproducibility and sensitivity [14]. However, one drawback, besides needing a separate ion chromatograph, is the complexity of the instrumentation, requiring membrane devices for proton‑potassium exchange and a carbonate remover. Therefore, the AIC system requires a highly trained operator. In contrast to reversed phase (RP)-LC, hydrophilic interaction liquid chromatography (HILIC) separates polar compounds according to their hydrophilic interaction with the stationary phase [15]. For HILIC analysis, the sample solution should contain more than 50% organic solvent (mostly acetonitrile), which decreases the solubility of polar compounds. This limits usage of aqueous solutions, in which sugar phosphates readily dissolve [16, 17]. Although in HILIC analysis it is difficult to separate isomeric sugar phosphates and it has been shown that for some metabolites, the reproducibility of HILIC is poor [18], there are reports where different sugar phosphates have been analyzed by HILIC chromatography [19, 20].

RP-LC is not suitable for highly polar compounds because they are not retained on a C_18_ column. To overcome this problem, different types of derivatization techniques have been used to improve retention in LC–MS. For example, 2-aminopyridine [21], *p*-amino benzoic ethyl ester [22] or 4-(3-methyl-5-oxo-2-pyrazolin-1-yl) benzoic acid [23] reagents have been used in different studies to improve chromatographic retention of highly polar compounds. However, these derivatization methods are tedious and not reliable due to incomplete derivatization. It has also been shown that derivatization of highly polar compounds by either propionylation or benzoylation can improve chromatographic retention in LC–ESI–MS [24].

In this study, we report on the suitability of derivatizing sugar phosphates with methoxylamine and propionic acid anhydride for identification and quantification of sugar phosphates using ultra-high-performance liquid chromatography–electrospray ionization–mass spectrometry (UHPLC–ESI–MS). We demonstrate the utility of this method on sugar phosphates extracted from *Populus* leaf and wood using a chloroform/methanol extraction.

## Results

### Separation and detection of standards by ultra-high performance liquid chromatography–electrospray ionization–mass spectrometry (UHPLC–ESI–MS)

**Fig. 1:**
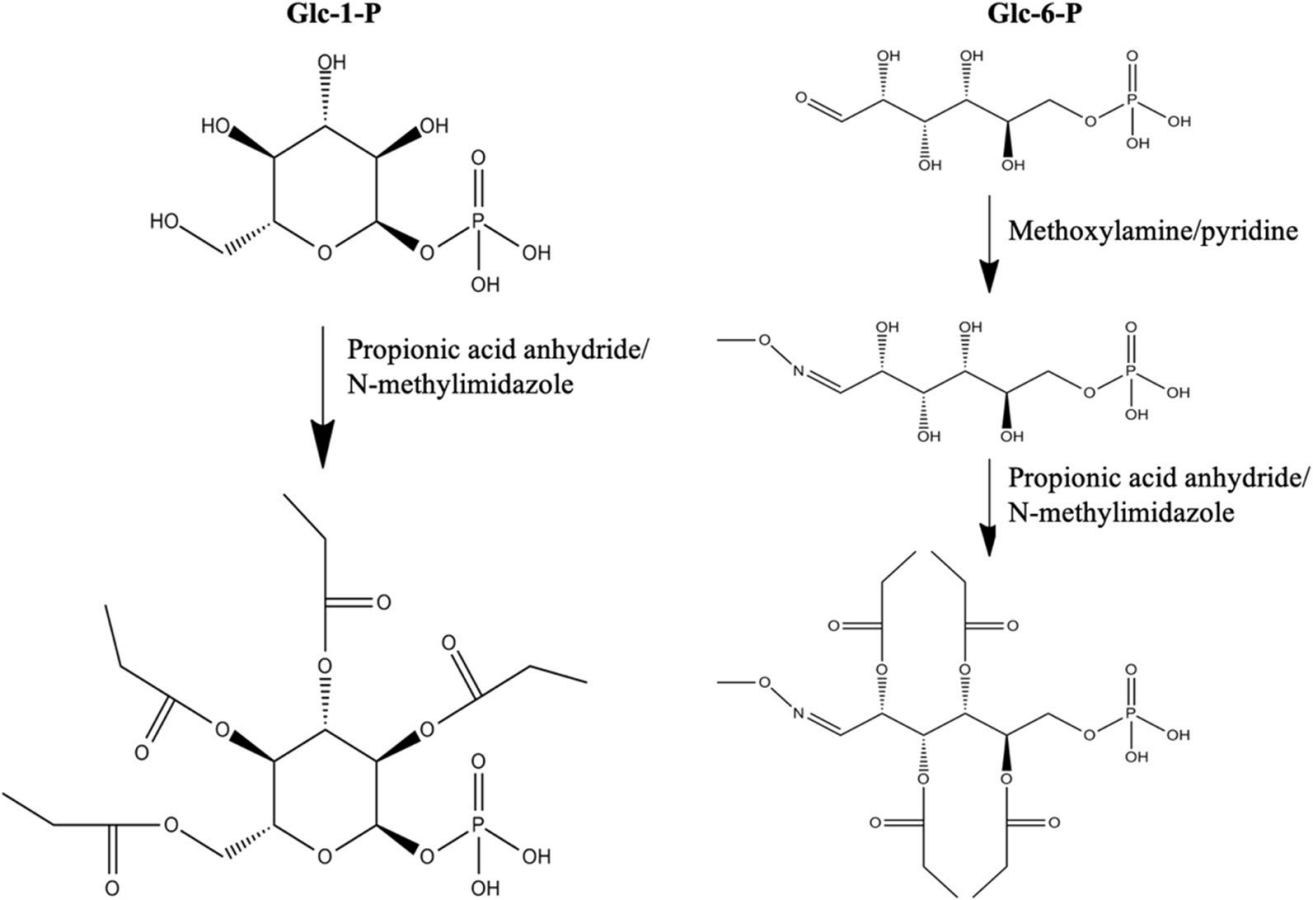
Examples of derivatized sugar phosphates. This figure illustrates propionylation of glucose-1-P and derivatization of glucose-6-P by methoxyamine/pyridine and methylimidazol/propionic anhydrate

To improve the chromatographic retention in reversed phase RP-LC, a two-step derivatization method was developed. Compounds with a free carbonyl group, e.g., glucose-6-phosphate, fructose-6-phosphate and 3-phosphoglyceraldehyde, were reacted to form methoxime derivatives (CH_3_ON) using methoxyamine [7] and then a second derivatization was conducted with propionic acid anhydride and *N*-methylimidazol as a catalyst to esterify hydroxyl groups by propionylation [24] (Fig. 1). Compounds without a free carbonyl group, e.g., glucose 1-phosphate or sucrose 6-phosphate, did not react with methoxylamine, only with propionic acid anhydride (Fig. 1).

**Fig. 2:**
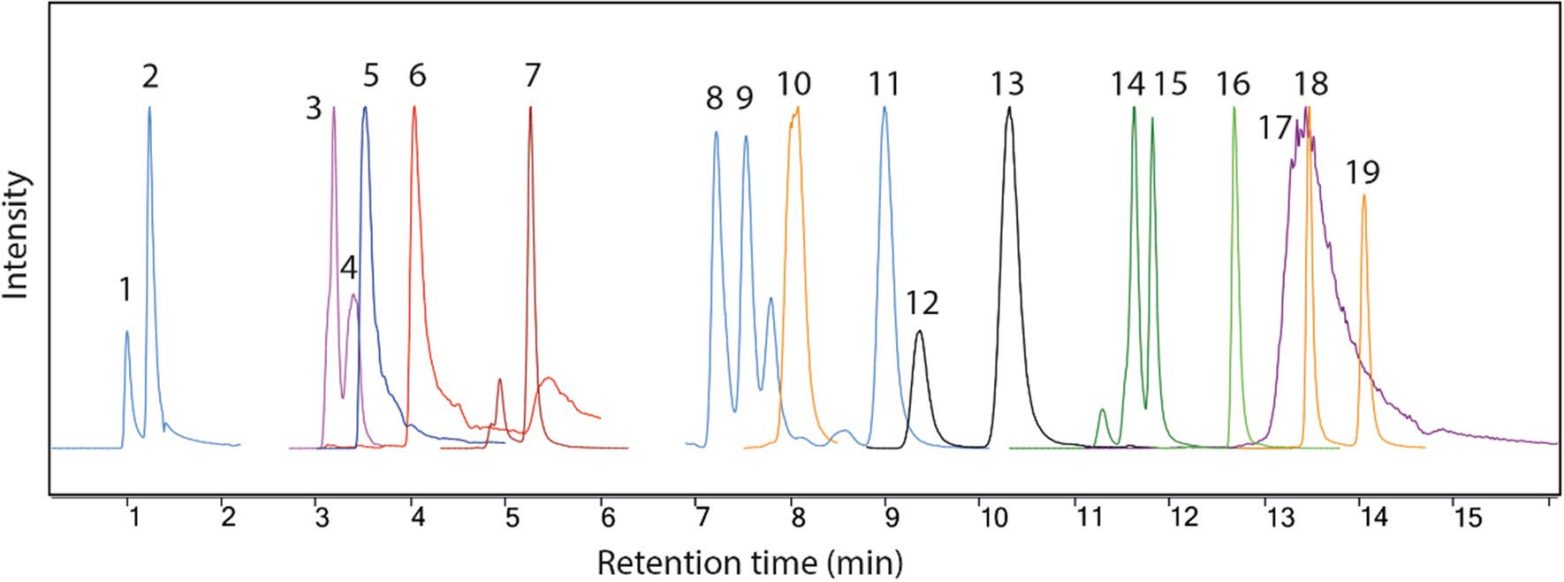
UHPLC-QqQ-MS MRM profiles of sugar phosphate derivatives of standard solutions consisting of: (1) 3-PGA, (2) 2-PGA, (3) GAP, (4) DHAP, (5) RuBP, (6) FBP, (7) E4P, (8) X5P, (9) Ru5P, (10) 2-DeoxyGP (IS), (11) R5P, (12) Gal1P, (13) G1P, (14) F6P, (15) G6P, (16) Sedu7P, (17) UDP-G, (18) T6P, (19) S6P

Nineteen standard compounds were derivatized and subsequently analyzed by UHPLC–ESI–MS. A known problem with analysis of sugar phosphates by RP-LC is peak tailing, which has been attributed to interactions between phosphate groups and the column [25]. The traditional way of reducing tailing in LC–UV analysis is to use phosphoric acid or phosphate buffers, but they are not compatible with MS detection due to low volatility. Therefore, to overcome this problem, different concentrations of formic acid (HCOOH) in the mobile phase were investigated (Additional file 1: Fig. S2). By increasing the concentration of formic acid, less tailing was observed for the majority of compounds. This was especially pronounced for bisphosphates and UDP-glucose. The optimized mobile phase (water/MeOH) contained 2% formic acid (pH for aqueous mobile phase approx. pH 2.0). The final chromatographic system was able to separate all major sugar phosphates, including structural isomers, such as 2PGA/3PGA, R5P/X5P/Ru5P and G6P/G1P/F6P (Fig. 2; Additional file 1: Table S1). UDP-glucose co-eluted with disaccharide-phosphates and still showed chromatographic tailing even at 2% formic acid (Additional file 1: Fig. S2).

The two step derivatization made it easier to distinguish structural isomers such as glucose 1-P and glucose 6-P based on molecular weight. MS/MS spectra (Additional file 1: Fig. S1) and MRM-transitions (Additional file 1: Table S1) of these isomers using QqQ-instrumentation for quantitative analysis were different. Overall, the analysis showed that derivatization of sugar phosphates by oximation and propionylation is an efficient method for improving the retention and separation of sugar phosphates in RP-LC by making polar compounds more hydrophobic.

### Identification of sugar phosphates in *Populus* leaf and wood extracts

Before using the separation method for quantitative analysis, sugar phosphates were identified in an extract of *Populus* wood. Eighteen sugar phosphates, including UDP-Glc, were identified in the *Populus* wood extract based on comparison of the retention times with MS/MS spectra of standard compounds (Table 1). Mass determination of the identified compounds showed mass errors ranging from 0.05 (0.2 ppm) to 6.1 mDa (28.9 ppm), which was considered acceptable for most analytes. The largest mass error was calculated for fructose 1,6-BP, possibly due to interfering substances in the corresponding chromatographic area, as observed in the total ion chromatogram.

**Table 1:** Identification of sugar phosphates in extracts of *Populus* wood

Compound	Derivative	Measured mass (Da), [M−H]^−^	Theoretical mass (Da), [M−H]^−^	Mass error (ppm)	Mass error (mDa)
3-Phosphoglyceric acid (3-PGA)	Prop	241.0117	241.0119	0.8	0.2
2-Phosphoglyceric acid (2-PGA)	Prop	241.0117	241.0119	0.8	0.2
3-Phosphoglyceraldehyde (GAP)	MeOx, Prop	254.0427	254.0435	3.2	0.8
Dihydroxyacetone phosphate (DHAP)	MeOx, Prop	254.0422	254.0435	5.1	1.3
Erythrose-4-P	MeOx, Prop	ND	ND	ND	ND
Ribulose-1,5-PP (Ru5BP)	MeOx, Prop	450.0702	450.0572	28.9	1.3
Fructose-1,6-PP (FBP)	MeOx, Prop	536.1001	536.094	11.4	6.1
Xylulose-5-P (X5P)	MeOx, Prop	426.1165	426.1171	1.4	0.6
Ribulose-5-P (Ru5P)	MeOx, Prop	426.1169	426.1171	0.5	0.2
Ribose-5-P (R5P)	MeOx, Prop	426.1170	426.1171	0.2	0.1
Galactose-1-P (Gal1P)	Prop	483.1277	483.1273	0.8	0.4
Glucose-1-P (G1P)	Prop	483.1272	483.1273	0.2	0.05
Fructose-6-P (F6P)	MeOx, Prop	512.1530	512.1539	1.8	0.9
Glucose-6-P (G6P)	MeOx, Prop	512.1528	512.1539	2.2	0.9
Mannose-6-P (Man6P)	MeOx, Prop	512.1525	512.1539	2.7	0.3
Seduheptulose-7-P (Sedu7P)	MeOx, Prop	598.1907	598.1906	0.2	0.1
UDP-glucose (UDP-G)	Prop	901.2089	901.205	4.3	3.9
Trehalose-6-P (T6P)	Prop	813.2574	813.2588	1.7	1.4
Sucrose-6-P (S6P)	Prop	813.2561	813.2588	3.3	2.7

Derivative denotes type of derivative as follows: MeOx, methoxime; Prop, propionyl. Erythrose-4-P was not conclusively identified by UHPLC-QTOF-MS, and therefore mentioned as non-detected (ND)

### Sensitivity, linearity and reproducibility with standards, *Populus* leaf and wood extracts

The sensitivity of the LC–MS method was determined by analysis of standard compounds. Table 2 shows limit of detection (LOD) values for the analyzed compounds, which ranged from low to high femtomoles. Standard mixtures from 50 pg/µl to 20 ng/µl were analyzed to obtain a calibration curve for each metabolite (Table 2). 2-Deoxy-glucose 6-P was chosen as an internal standard (IS) since it is not considered an endogenous compound in plant extracts. The majority of analytes showed good linearity (*R*^2^ ≥ 0.99). Values for 3-PGA (*R*^2^ = 0.98) and UDP-G (*R*^2^ = 0.97) were also judged to be acceptable. These results indicate a good quantitative relationship between the MS response and analyte concentration. Moreover, most analytes were detected in the 50-5000 pg range. The dynamic range covered the concentration ranges of all detected analytes in the plant extracts examined in the study. The precision for standards was assessed by determining relative standard deviation (RSD, %) values of 5 replicate measurements: RSD values were < 9.0 for both the 125 pg and 2500 pg calibration levels.

**Table 2:** Lower limit of detection (LLOD), linearity, precision for two standard concentrations (*n *= 5) and method reproducibility for 5 samples of *Populus* leaf and wood tissue

Compound	LLOD (pmol)	Linearity range pg; R^2^	125 pg (%RSD)	2500 pg (%RSD)	*Populus* leaf (%RSD)	*Populus* wood (%RSD)
3-Phosphoglyceric acid	0.067	50–10,000; 0.98	6.9	4.1	7.6	16.4
2-Phosphoglyceric acid	0.007	50–10,000; 0.99	8.7	8.1	14.8	22.7
3-Phosphoglyceraldehyde	0.011	50–10,000; 0.99	5.7	3.5	14.7	7.8
Dihydroxyacetone phosphate	0.022	50–20,000; 0.99	4.9	3.6	7.7	6.1
Erythrose-4-P	0.045	50–5000; 0.99	6.4	4.2	10.7	29.8
Ribulose-5-PP	0.091	250–20,000; 0.99	na	4.3	12.6	8.1
Fructose-1,6-PP	0.25	500–20,000; 0.99	na	5.6	14.3	7.7
Xylulose-5-P	0.054	50–5000; 0.99	6.6	7.4	17.9	20.2
Ribulose-5-P	0.054	50–5000; 0.99	4.2	2.1	5.9	8.9
Galactose-1-P	0.029	50–5000; 0.99	7.4	2.5	11.2	4.8
Ribose-5-P	0.026	50–5000; 0.99	4.6	3.1	16.7	7.8
Glucose-1-P	0.024	50–5000; 0.99	1.6	2.2	18.9	11.5
Fructose-6-P	0.012	50–20,000; 0.99	1.5	1.8	7.8	3.5
Glucose-6-P	0.005	50–20,000; 0.99	3.4	2.6	5.7	4.1
Seduheptulose-7-P	0.008	125–20,000; 0.99	2.9	1.7	14.5	9.7
Trehalose-6-P	0.009	50–20,000; 0.99	5.7	1.9	16.2	11.0
Sucrose-6-P	0.011	50–5000; 0.99	6.6	2.9	21.1	17.8
UDP-glucose	0.074	125–10,000; 0.97	6.5	4.7	8.7	8.1

*%RSD* relative standard deviation in percentage

The reproducibility of the method was tested with 10 replicates of pooled extracts of *Populus* leaf and wood. According to the calculated RSD values (Table 2), the majority of the sugar phosphates had RSD 5-20% and 5-15% in *Populus* leaf and wood, respectively. S6P had RSD of 21.1% in leaf and 2-PGA, E4P and X5P had RSD values > 20% in wood. These high RSD values can be explained by low abundance and/or interference from closely co-eluting isomeric compounds (see e.g., Additional file 1: Fig. S2 S6P). The chemical stability of the extract was also determined by repeating the analysis of samples after 2 days in an autosampler at 5 °C. There were no significant differences in peak areas after 2 days, except for FBP, which showed > 75% reduction in peak area (data not shown).

### Quantification of sugar phosphates in *Populus* leaf and wood extracts, and recovery of standards during extraction

To quantify extracted sugar phosphates from *Populus* leaf and wood, UHPLC–ESI–QqQ-MS was used. The analysis quantified sugar phosphates as ranging between 1.8 and 86.2 ng/mg in leaves and 2.0–143.2 ng/mg in wood samples (Table 3). However, S6P in leaf samples and 3-PGA, 2-PGA, G1P, S7P and S6P in wood samples could not be quantified because their concentrations were outside the limit of the calibration curve, and therefore below the lower limit of detection. However, they could be quantified by extending the calibration curves and by lowering the concentration of IS in the samples.

**Table 3:** Quantification of sugar phosphates in samples of *Populus* leaf and wood by UHPLC-QqQ-MS

Compound	ng/mg FW	
	*Populus* leaf	*Populus* wood
3-Phosphoglyceric acid	52.4 ± 4.0	nq
2-Phosphoglyceric acid	2.6 ± 0.4	nq
3-Phosphoglyceraldehyde	2.4 ± 0.4	2.5 ± 0.2
Dihydroxyacetone phosphate	4.0 ± 0.3	2.0 ± 0.1
Erythrose-4-P	1.8 ± 0.3	2.0 ± 0.2
Ribulose-5-PP	27.6 ± 3.8	27.0 ± 2.4
Fructose-1,6-PP	25.6 ± 4.0	28.7 ± 2.4
Xylulose-5-P	7.1 ± 1.4	7.2 ± 1.6
Ribulose-5-P	7.9 ± 0.5	6.7 ± 0.7
Galactose-1-P	18.0 ± 2.2	19.1 ± 1.0
Ribose-5-P	11.9 ± 2.2	13.3 ± 1.1
Glucose-1-P	1.9 ± 0.5	nq
Fructose-6-P	27.0 ± 2.3	25.3 ± 1.0
Glucose-6-P	65.4 ± 4.1	58.9 ± 2.7
Seduheptulose-7-P	24.2 ± 3.9	nq
Trehalose-6-P	3.6 ± 0.6	4.2 ± 0.5
Sucrose-6-P	nq	nq
UDP-glucose	86.2 ± 7.5	143.2 ± 11.7

*nq* not quantified

Recovery tests were performed to investigate whether the metabolites of interest were recovered during chloroform/methanol (3:7) extraction. The 8 standard compounds including the IS with known amounts (Table 4) were derivatized either with or without performing the extraction procedure. Calculations revealed that the recovery of sugar phosphates by chloroform/methanol (3:7) extraction ranged between 70 and 92%, except for 3-PGA (63.5%) (Table 4). The results showed that there were losses during the extraction procedure, probably because the two-phase extraction procedure involved organic/aqueous solvent partitioning. Alternatively, a one-phase extraction method could have been used [7], but this method needs to be validated for analysis of sugar phosphates.

**Table 4:** Recovery of sugar phosphate standards after extraction and derivatization

Compound	% recovery
3-PGA	63.5
Fructose-1,6-PP	84.9
2-Deoxyglucose (IS)	76.9
Glucose 1-P	70.5
Glucose 6-P	76.3
Fructose 6-P	69.5
Trehalose 6-P	84.5
UDP-glucose	92.1

To determine the accuracy of quantitation, metabolites were extracted from *Populus* leaf and analytical recoveries of extracted samples were measured by a two-level spiking approach of selected metabolites. The spiking levels were chosen as they correspond to medium and high levels of the metabolites in the samples. The results after correcting the signals according to the IS showed that the accuracy in analyzing *Populus* leaf extracts was good (79–107%) for all selected metabolites, even for small quantities of G1P and T6P (Table 5). The recovery of the IS in the samples was between 60 and 75%. The accuracy and recovery tests indicated that all the selected metabolites were extracted quantitatively from plant material, and quantification of the metabolites by the derivatization-LC/MS method was accurate and robust.

**Table 5:** Accuracy of UHPLC-QqQ-MS analysis of selected sugar phosphates spiked in *Populus* leaf extract (mean ± SD, *n *= 5)

Compound	Added quantity (ng)	Accuracy (%)	Added quantity (ng)	Accuracy (%)
3-Phosphoglyceric acid	50	88 ± 8	150	95 ± 6
Fructose 1,6-BP	20	79 ± 14	60	88 ± 12
Glucose 1-P	40	102 ± 6	120	107 ± 9
Glucose 6-P	500	105 ± 8	150	91 ± 10
Fructose 6-P	65	92 ± 10	195	89 ± 8
Trehalose 6-P	85	88 ± 9	255	97 ± 7
UDP-glucose	690	85 ± 7	2070	86 ± 12

Intriguingly, we also detected two unknown disaccharide phosphates with retention times between those of T6P and S6P (Additional file 1: Fig. S3). The tandem mass spectra were identical for the different isomers and as no other standards were available, we were not able to annotate the two closely related peaks. According to the derivatization strategy, the unknown compounds are likely to be disaccharide phosphates of a non-reducing sugar or a reducing sugar with the phosphate group in the 1-position, such as in maltose-1-phosphate.

## Discussion

Sugar phosphates have diverse physicochemical properties. Chemical analysis of these metabolites is challenging due to their poor chromatographic retention in traditional RP-LC. In this study, we improved the chromatographic properties of sugar phosphates and related compounds on RP-LC by utilizing a two-step derivatization method compatible with both RP-LC and MS.

Initially, the chromatographic separation was investigated on different RP-C_18_ UHPLC columns, such as the Waters Acquity HSS-T3, Waters Acquity BEH-C18, Phenomenex Kinetex EVO-C_18_, Phenomenex Kinetex Biphenyl, Phenomenex Kinetex phenyl-hexyl, Phenomenex Kinetex pentaflurophenyl and Phenomenex Kinetex Synergi Fusion-RP C18 columns. Mobile phases consisting of water (with 0.1–2% HCOOH) and MeOH or AcN (with 0.1–2% HCOOH) with binary gradient elution were used. Low pH can affect the stability of traditional silica based reverse phase columns, hence no more then 2% of formic acid was used in this study. The best separation and least tailing were achieved with the Waters Acquity HSS-T3, Phenomenex Kinetex EVO C_18_ or Waters Acquity BEH-C18 column, and MeOH as organic mobile solvent. A pentafluorophenyl core shell UHPLC column has previously been shown to give good results for some 3-amino-9-ethylcarbazole derivatized selected sugar phosphates [26] but did not improve peak separation in the present study.

The derivatization of metabolites can cause some degradation of analytes and low derivatization efficiencies, which can affect results. However, we did not detect any underivatized analytes during the analysis of derivatized standard compounds by UHPLC-QTOF-MS (data not shown). Instead, the analysis showed that derivatization of sugar phosphates by oximation and propionylation is an efficient method for improving the retention and separation of sugar phosphates in RP-LC by making polar compounds more hydrophobic.

An important issue in plant metabolite analysis is the choice of extraction method. Since the metabolome is dynamic, a fast and simple sampling technique is required to quench metabolic activity. This is especially important for compounds like sugar phosphates, which are easily affected by activity of phosphatases and kinases during extraction procedures. In this study, two different extraction protocols were tested. The first was a metabolomics protocol described in [7], which is a single phase extraction with a chloroform/MeOH/H_2_O (1:3:1) mixture. The second protocol was extraction with chloroform/methanol (3:7) and partitioning against H_2_O as described in [6]. Although only small differences were detected between these two extraction protocols, the widely accepted method using chloroform/methanol (3:7) was chosen for further analysis.

The sensitivity of the LC–MS method was determined by analysis of standard compounds. The derivatization was found to have a positive effect on sensitivity, with many compounds showing 10 to 100 times improvement compared to other published methods [6, 12, 13, 27, 28]. In addition, the method showed a similar or better sensitivity compared to the reductive amination method of reducing sugars used by Han et al. [26].

The accuracy and precision depends on the choice of IS. In our study, we only used 2-deoxy-glucose 6-P as IS. In order to cover a wide range of metabolites, additional ISs, preferably stable isotope labeled ones, could be included in the analysis. Another consideration in this study was the purification of extracts prior to derivatization. We tested solid-phase extraction (SPE) purification prior to the derivatization, but it did not improve the quality of the analysis (data not shown). However, a drawback of the derivatization strategy, which was evident in UHPLC-QTOF-MS analysis of derivatization blank injections, was an increase in background noise caused by the derivatization reagents. Therefore, SPE purification after derivatization or on-line SPE UHPLC-QqQ-MS analysis may enable further improvement of the method.

Previous studies using AEC–MS/MS [6] or IPC–MS/MS [12, 13] were not able to detect or separate disaccharide phosphates together with monosaccharide phosphates. In our study, similar to AIC–MS/MS analysis [14], we were able to separate and detect not only T6P/S6P isomers (Additional file 1: Fig. S3) but also hexose-1-P/hexose-6-P isomers (Additional file 1: Fig. S4) in *Populus* wood extract. Protocols for the analysis of derivatized sugar phosphates have been published [9, 26], but to the best of our knowledge, this is the first time that a derivatization strategy has also allowed simultaneous analysis of hexose-1-P/hexose-6-P isomers.

## Conclusion

Here, we report the development of a method combining a two-step derivatization procedure with LC–MS for quantitative analysis of sugar phosphates in plant extracts. The major advantage of the methodology is that reversed chromatography can be used without the need for addition of ion-pairing reagents to the mobile phase, thereby eliminating problems caused by ion-pairing reagents. This study represents the first evaluation of chemical derivatization of sugar phosphates where both reducing and non-reducing sugar phosphates can be analyzed by RP–LC–MS. The chemical derivatization strategy facilitates separation of structural isomers, such as hexose 1-phosphates and hexose 6-phosphates, which otherwise is challenging. The derivatization not only improved the chromatographic behavior on a RP column but also increased the sensitivity in negative ion ESI–MS mode. Although the focus of this study was sugar phosphates, the method could also be used for organic acids and reducing sugars. Thus, the method could be applied to a complete set of metabolites involved in energy metabolism in plants and other organisms.

## Methods

### Chemicals

All metabolite standards and other chemicals were purchased from Sigma-Aldrich (Minneapolis, MN, USA), except sedoheptulose-7-phosphate, which was purchased from Carbosynth Limited (Berkshire, UK). All standards were purchased at the highest available purity.

### Plant material and growth conditions

*Populus* wood samples were obtained from developing wood of field-grown 7-year-old aspen (*Populus tremula)* trees. Leaf samples were obtained from the 5th leaf counted from the top of 2-month-old hybrid aspen (*Populus tremula x tremuloides)* trees that had been grown in a greenhouse in a commercial soil/sand/fertilizer mixture (Yrkes Plantjord; Weibulls Horto) at 20/15 °C (light/dark) with a 18 h light/6 h dark photoperiod. The greenhouse grown trees were fertilized using approximately 150 ml 1% Rika-S (N/P/K 7:1:5; Weibulls Horto) once a week after 3 weeks of planting. The harvested samples were flash frozen in liquid N_2_ immediately and stored at − 80 °C. Developing wood was obtained by peeling the bark and scraping the exposed wood using a scalpel. Both developing wood and leaf samples were ground in liquid N_2_ using a pestle and mortar and then stored in a − 80 °C freezer until extraction.

### Metabolite extraction and derivatization

10 mg of each sample was placed into 1.5 ml Eppendorf tubes together with 250 µl of ice-cold extraction medium (chloroform/methanol, 3:7) and incubated at − 20 °C for 2 h. Afterwards, 194 ng of 2-deoxy-d-glucose 6-phosphate was added to each sample as an IS. Samples were then extracted twice with 200 µl of ice-cold water and the aqueous layers were pooled and dried by a freeze-dryer [6].

For derivatization, dried samples were dissolved in 20 µl of methoxylamine and incubated on a heat block at 60 °C for 30 min. After incubation at room temperature overnight [7], 6 µl 1-methylimidazol and 12 µl propionic acid anhydride were added. The reaction mixture was heated at 37 °C for 30 min and then evaporated to dryness using N_2_ gas. Prior to LC–MS analysis, derivatized metabolites were dissolved in 100 µl of aqueous 0.1% formic acid.

### Preparation of standards

A calibration curve was prepared using eight different concentrations (from 50 pg/µl to 20 ng/µl) of standards, which are listed in Table 2. Five replicates were used for each calibration level.

### Test of derivatization reproducibility, accuracy and recovery

For the reproducibility test, *Populus* wood and leaf samples obtained from 10 biological replicates were pooled. Next, 10 mg of the pooled samples were used for each analysis with 5 replicates. The accuracy test was carried out by adding 1× and 3× concentrations of standard mixtures to the samples, followed by freeze drying. The standard mixture consisted of 500 ng glucose-6-P, 40 ng glucose-1-P, 65 ng fructose-6-P, 690 ng UDP-glucose, 20 ng fructose-1,6-P, 85 ng trehalose-6-P and 50 ng 3-phosphoglyceric acid. The recovery test was carried out by comparing extracted/derivatized IS + standard mixture with only derivatized IS + standard mixture.

RSD values were calculated to evaluate the reproducibility and accuracy of both the derivatization and detection method. Lower limits of detection (LOD) was calculated as the minimum concentration injected that gave a detector response higher than three times the signal-to-noise ratio (S/N) [29]. Besides determine the noise level by averaging the results from 5 repeated injections, the calculation was based on the slope of the calibration curve.

### LC–MS analysis

Quantitative LC–MS analysis was performed on an Agilent 1290 Infinity UHPLC system coupled with an Agilent 6495 QqQ-MS (Agilent Technologies, Santa Clara, CA, USA) operated in the dynamic multiple-reaction-monitoring (MRM) mode. Chromatographic separation was performed on a Waters Acquity HSS-T3 1.7 µm, 2.1 × 50 mm column (Waters Corporation, Milford, USA). The washing solution used in the autosampler syringe and injection needle comprised 90% MeOH with 1% HCOOH. The mobile phase A consisted of 2% HCOOH and B MeOH with 2% HCOOH with a pH approx. pH 2.0. The following gradient was used: 0.1% B for 1 min, followed by linear gradients from 0.1 to 30% from 1 to 3 min, then 30 to 40% B from 3 to 6 min, hold at 40% B from 6 to 10 min, followed by 40 to 70% B from 10 to 12.5 min, hold at 70% B from 12.5 to 15 min, and then 70 to 99% B from 15 to 17 min hold at 99% B for 0.5 min, and thereafter the column was re-equilibrated to 0.1% B. The flow rate was 0.8 ml min^−1^ during equilibration and 0.5 ml min^−1^ during the chromatographic runs. The column was heated to 40 °C, and the injection volumes were 1 μl. The mass spectrometer was operated in negative ESI mode with the following settings: gas temperature 230 °C, gas flow 12 l min^−1^, nebulizer pressure 20 psi, sheath gas temperature 400 °C, sheath gas flow 12 l min^−1^, capillary voltage 4000 V (neg), nozzle voltage 500 V, iFunnel high pressure RF 150 V, iFunnel low pressure RF 60 V, fragmentor voltage 380 V, cell acceleration voltage 5 V. For a list of MRM transitions, see Additional file 1: Table S1. Data were processed using MassHunter Qualitative Analysis and Quantitative Analysis (QqQ; Agilent Technologies, Santa Clara, CA, USA) software.

Identification of sugar phosphates was performed on a 1290 Infinity system from Agilent Technologies (Waldbronn, Germany) with an Agilent 6550 QTOF mass spectrometer (Agilent Technologies, Santa Clara, CA, USA) for MS detection. The chromatography system was as described above. The QTOF mass spectrometer was equipped with a jet stream electrospray source operating in negative ion mode. The capillary voltage was set at 3.5 kV. The jet-stream gas temperature was 150 °C with a gas flow rate of 16 l min^−1^, sheath gas temperature of 350 °C and sheath gas flow rate of 11 l min^−1^. The nebulizer pressure was set to 35 psi. The fragmentor voltage was 380 V and the collision energy was set at 0 V when the MS mode was applied. The mass range of the TOF was set to 70–1700 *m*/*z* with a scan rate of 4 scans s^−1^. Purine (2 µM) and HP-0921 (hexakis(1H, 1H, 3H-tetrafluoropropoxy)phosphazine) (2.5 µM), both purchased from Agilent Technologies (Santa Clara, CA, USA), were used as reference masses and infused using an isocratic pump with a 1.9 mL/min flow rate and 1:100 split. The resolution was about 20,000. For tandem mass spectra analysis, the collision energy was set between 10 and 60 V. MSMS spectra were obtained in targeted mode with parent ion selection of 1.2 amu. Product ion mass spectra were obtained over the mass range 40–1000 *m*/*z* at a scan range of 3 scans s^−1^.

## Supplementary information

**Additional file 1.** Additional figures and table.

## Data Availability

All data generated or analyzed during this study are included in this published article (and its additional file).

## References

[1] Oliver SG, Winson MK, Kell DB, Baganz F (1998). Systematic functional analysis of the yeast genome. Trends Biotechnol.

[2] Kruger NJ, von Schaewen A (2003). The oxidative pentose phosphate pathway: structure and organisation. Curr Opin Plant Biol.

[3] Bieleski RL (1964). The problem of halting enzyme action when extracting plant tissues. Anal Biochem.

[4] Jelitto T, Sonnewald U, Fau-Willmitzer L, Willmitzer L, Fau-Hajirezeai M, Hajirezeai M, Fau-Stitt M, Stitt M (1992). Inorganic pyrophosphate content and metabolites in potato and tobacco plants expressing *E. coli* pyrophosphatase in their cytosol. Planta.

[5] Johansen HN, Glitsø V, Bach Knudsen KE (1996). Influence of extraction solvent and temperature on the quantitative determination of oligosaccharides from plant materials by high-performance liquid chromatography. J Agric Food Chem.

[6] Lunn JE, Feil R, Hendriks JH, Gibon Y, Morcuende R, Osuna D (2006). Sugar-induced increases in trehalose 6-phosphate are correlated with redox activation of ADPglucose pyrophosphorylase and higher rates of starch synthesis in *Arabidopsis thaliana*. Biochem J.

[7] Gullberg J, Jonsson P, Nordstrom A, Sjostrom M, Moritz T (2004). Design of experiments: an efficient strategy to identify factors influencing extraction and derivatization of *Arabidopsis thaliana* samples in metabolomic studies with gas chromatography/mass spectrometry. Anal Biochem.

[8] Soga T, Igarashi K, Ito C, Mizobuchi K, Zimmermann H-P, Tomita M (2009). Metabolomic profiling of anionic metabolites by capillary electrophoresis mass spectrometry. Anal Chem.

[9] Okahashi N, Maeda K, Kawana S, Iida J, Shimizu H, Matsuda F (2019). Sugar phosphate analysis with baseline separation and soft ionization by gas chromatography-negative chemical ionization-mass spectrometry improves flux estimation of bidirectional reactions in cancer cells. Metab Eng.

[10] Bennett RN, Mellon FA, Kroon PA (2004). Screening crucifer seeds as sources of specific intact glucosinolates using ion-pair high-performance liquid chromatography negative ion electrospray mass spectrometry. J Agric Food Chem.

[11] Coulier L, Bas R, Jespersen S, Verheij E, van der Werf MJ, Hankemeier T (2006). Simultaneous quantitative analysis of metabolites using ion-pair liquid chromatography–electrospray ionization mass spectrometry. Anal Chem.

[12] Luo B, Groenke K, Takors R, Wandrey C, Oldiges M (2007). Simultaneous determination of multiple intracellular metabolites in glycolysis, pentose phosphate pathway and tricarboxylic acid cycle by liquid chromatography-mass spectrometry. J Chromatogr A.

[13] Arrivault S, Guenther M, Ivakov A, Feil R, Vosloh D, van Dongen JT (2009). Use of reverse-phase liquid chromatography, linked to tandem mass spectrometry, to profile the Calvin cycle and other metabolic intermediates in *Arabidopsis rosettes* at different carbon dioxide concentrations. Plant J..

[14] Schwaiger M, Rampler E, Hermann G, Miklos W, Berger W, Koellensperger G (2017). Anion-exchange chromatography coupled to high-resolution mass spectrometry: a powerful tool for merging targeted and non-targeted metabolomics. Anal Chem.

[15] Hemstrom P, Irgum K (2006). Hydrophilic interaction chromatography. J Sep Sci.

[16] Jandera P (2011). Stationary and mobile phases in hydrophilic interaction chromatography: a review. Anal Chim Acta.

[17] Johnson JR, Karlsson D, Dalene M, Skarping G (2010). Determination of aromatic amines in aqueous extracts of polyurethane foam using hydrophilic interaction liquid chromatography and mass spectrometry. Anal Chim Acta.

[18] Bajad SU, Lu W, Kimball EH, Yuan J, Peterson C, Rabinowitz JD (2006). Separation and quantitation of water soluble cellular metabolites by hydrophilic interaction chromatography–tandem mass spectrometry. J Chromatogr A.

[19] Antonio C, Larson T, Gilday A, Graham I, Bergstrom E, Thomas-Oates J (2008). Hydrophilic interaction chromatography/electrospray mass spectrometry analysis of carbohydrate-related metabolites from *Arabidopsis thaliana* leaf tissue. Rapid Commun Mass Spectrom RCM..

[20] Schatschneider S, Abdelrazig S, Safo L, Henstra AM, Millat T, Kim DH (2018). Quantitative isotope-dilution high-resolution-mass-spectrometry analysis of multiple intracellular metabolites in *Clostridium autoethanogenum* with uniformly (13)C-labeled standards derived from Spirulina. Anal Chem.

[21] Hase S, Ikenaka T, Matsushima Y (1978). Structure analyses of oligosaccharides by tagging of the reducing end sugars with a fluorescent compound. Biochem Biophys Res Commun.

[22] Kwon H, Kim J (1993). Determination of monosaccharides in glycoproteins by reverse-phase high-performance liquid chromatography. Anal Biochem.

[23] Castells CB, Arias VC, Castells RC (2002). Precolumn derivatization of reducing carbohydrates with 4-(3-Methyl-5-oxo-2-pyrazolin-1-yl) benzoic acid. Study of reaction, high-performance liquid chromatographic separation and quantitative performance of method. Chromatographia..

[24] Nordstrom A, Tarkowski P, Tarkowska D, Dolezal K, Astot C, Sandberg G (2004). Derivatization for LC-electrospray ionization-MS: a tool for improving reversed-phase separation and ESI responses of bases, ribosides, and intact nucleotides. Anal Chem.

[25] Wakamatsu A, Morimoto K, Shimizu M, Kudoh S (2005). A severe peak tailing of phosphate compounds caused by interaction with stainless steel used for liquid chromatography and electrospray mass spectrometry. J Sep Sci.

[26] Han J, Tschernutter V, Yang J, Eckle T, Borchers CH (2013). Analysis of selected sugars and sugar phosphates in mouse heart tissue by reductive amination and liquid chromatography–electrospray ionization mass spectrometry. Anal Chem.

[27] Cruz JA, Emery C, Wust M, Kramer DM, Lange BM (2008). Metabolite profiling of Calvin cycle intermediates by HPLC-MS using mixed-mode stationary phases. Plant J..

[28] Antonio C, Larson T, Gilday A, Graham I, Bergström E, Thomas-Oates J (2007). Quantification of sugars and sugar phosphates in *Arabidopsis thaliana* tissues using porous graphitic carbon liquid chromatography–electrospray ionization mass spectrometry. J Chromatogr A.

[29] ICH (2015). ICH harmonised tripartite guideline: validation of analytical procedures: text and methodology, Q2(R1).

