# Supplementary material for: Two-step derivatization for determination of sugar phosphates in plants by combined reversed phase chromatography/tandem mass spectrometry
Source: Plant Methods. 2019 Nov 7;15:127. doi: 10.1186/s13007-019-0514-9 (PMC6836659; doi:10.1186/s13007-019-0514-9)
Supplement: Supplementary file 1 — Additional file 1. Additional figures and table. [file 13007_2019_514_MOESM1_ESM.docx]

## Additional file 1


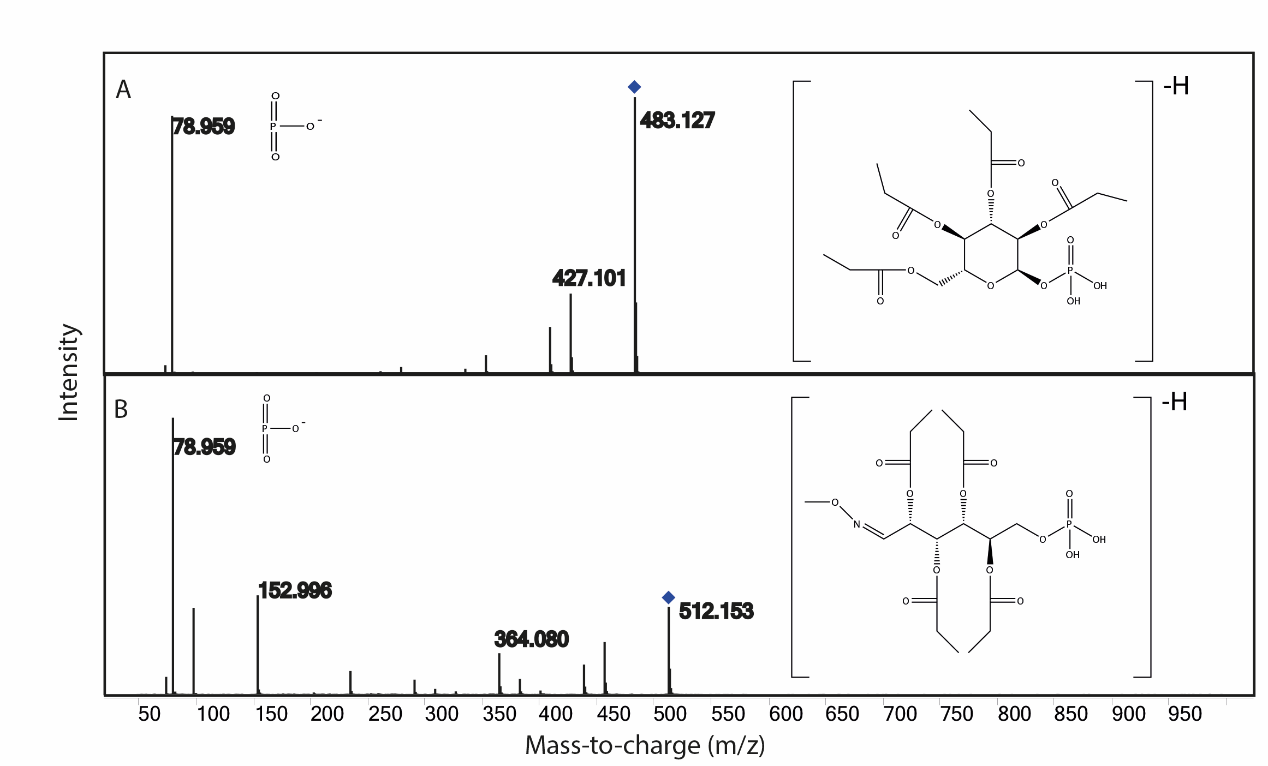


**Figure S1**. MS/MS spectra for derivatized glucose-6-phosphate ([M-H]^-^ = 512) and glucose-1-P ([M-H]^-^ = 483)


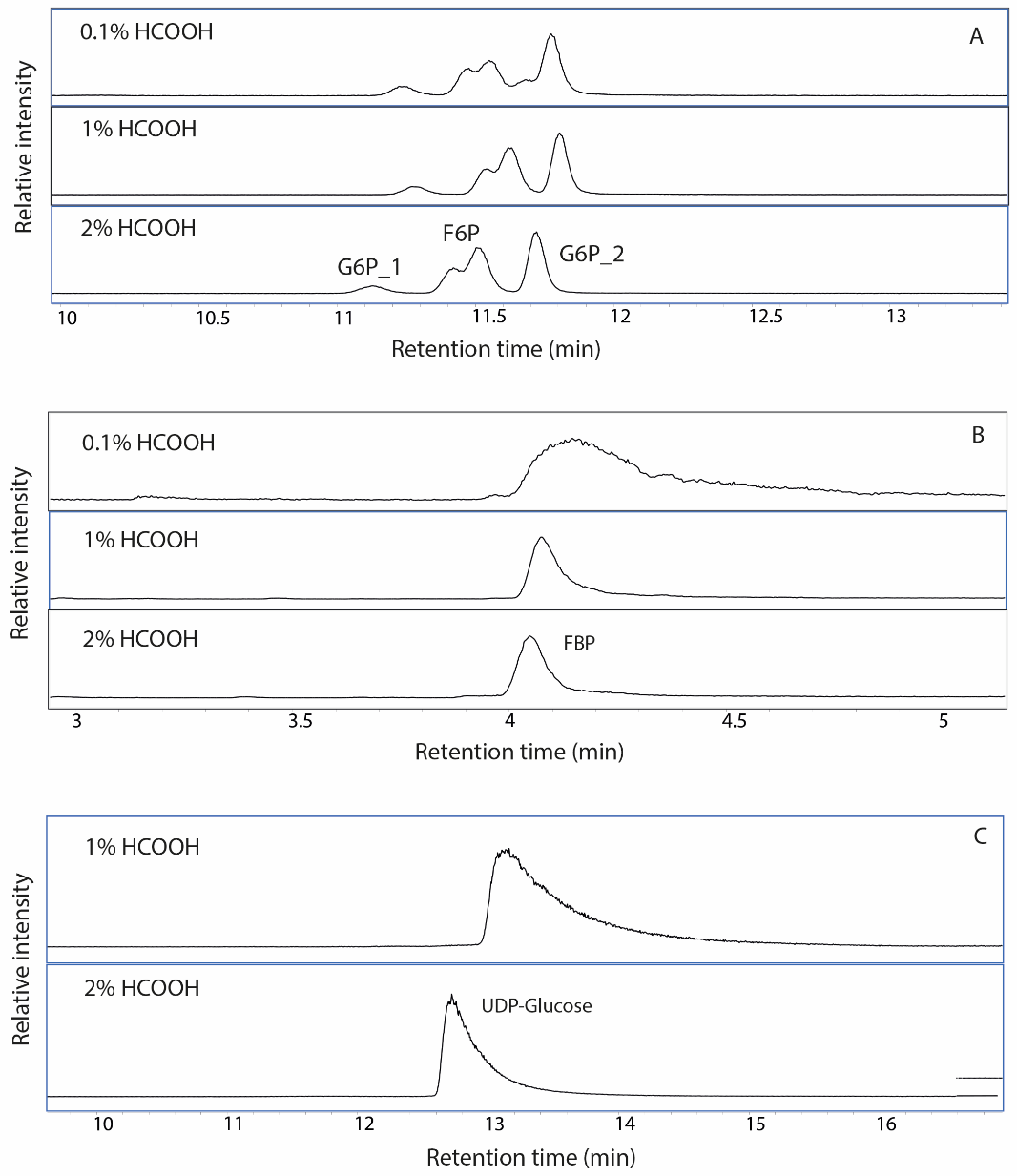


**Figure S2.** **Comparison** of chromatographic separation of (A) G6P and F6P, (B) FBP and (C) UDP-G with different concentrations of HCOOH in the mobile phase.


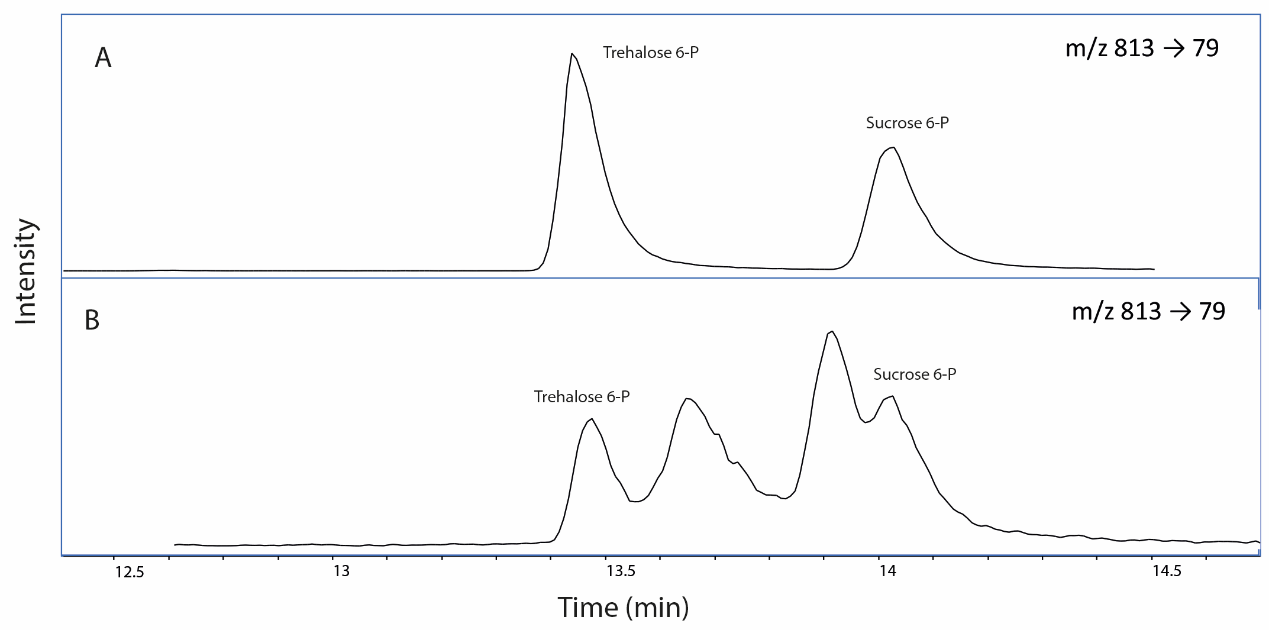


**Figure S3.** UHPLC-QqQ-MS MRM profiles of (A) T6P and S6P standard compounds **and** (B) extract from *Populus* wood.


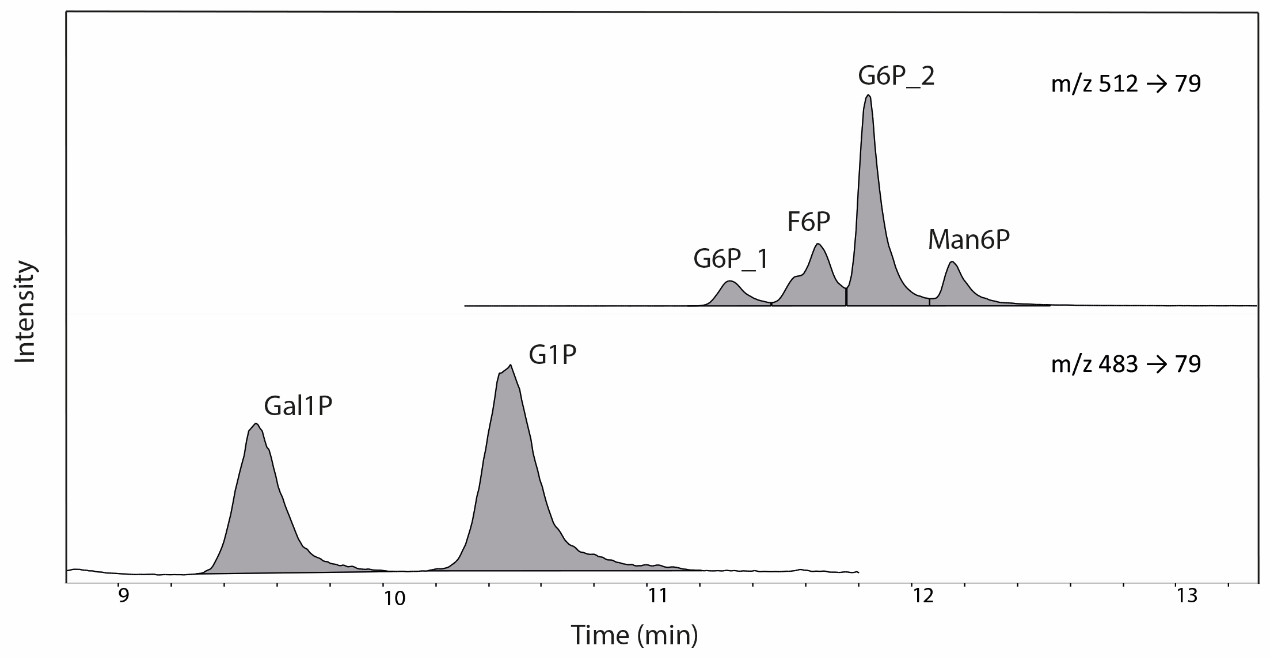


**Figure S4.** UHPLC-QqQ-MS MRM profiles of hexose 6-P and hexose 1-P in extract from *Populus* wood.

**Table S1.** All MRM transitions for UHPLC-QqQ-MS analysis. RT=retention time; CE = **collision energy**.

| **Compound^1^** | **RT (min)** | **Quantifier** | **CE** | **Qualifier** | **CE** |
| --- | --- | --- | --- | --- | --- |
| 3-phosphoglyceric acid | 0.9 | 241→79 | 50 | 241→151 | 5 |
| 2-phosphoglyceric acid | 1.1 | 241→79 | 50 | 241→151 | 5 |
| 3-phophoglyceraldehyde | 3.4 | 254→79 | 50 | 254→180 | 5 |
| Dihydroxyacetone phosphate | 3.2 | 254→79 | 50 | 254→180 | 5 |
| Ribulose-5-PP | 3.7 | 450→79 | 50 | 450→159 | 17 |
| Fructose-1,6-PP | 4.2 | 536→79 | 50 | 536→159 | 21 |
| Erythrose-4-P | 5.3 | 340→79 | 50 | 340→266 | 5 |
| Xylulose-5-P | 7.35 | 426→79 | 49 | 426→37 | 9 |
| Ribulose-5-P | 7.65 | 426→79 | 49 | 426→37 | 9 |
| 2-Deoxyglucose 6-P (IS) | 8.2 | 440→79 | 41 | - | - |
| Ribose-5-P | 9.1 | 426→79 | 49 | 426→37 | 9 |
| Galactose-1-P | 9.5 | 483→79 | 37 | 483→427 | 13 |
| Glucose-1-P | 10.5 | 483→79 | 37 | 483→427 | 13 |
| Fructose-6-P | 11.6 | 512→79 | 37 | 512→153 | 13 |
| Glucose-6-P | 11.8 | 512→79 | 37 | 512→153 | 13 |
| Seduheptulose-7-P | 12.7 | 598→79 | 49 | 598→542 | 17 |
| UDP-glucose | 13.5 | 901→497 | 41 | 901→545 | 37 |
| Trehalose-6-P | 13.5 | 813→79 | 50 | 813→757 | 25 |
| Sucrose-6-P | 14.1 | 813→79 | 50 | 813→757 | 25 |
